# One size does not fit all: an exploratory interview study on how translational researchers navigate the current academic reward system

**DOI:** 10.3389/fmed.2023.1109297

**Published:** 2023-05-05

**Authors:** Farah R. W. Kools, Christine M. Fox, Berent J. Prakken, Harold V. M. van Rijen

**Affiliations:** Center of Education and Training, University Medical Center Utrecht, Utrecht University, Utrecht, Netherlands

**Keywords:** translational research, academic reward system, publication pressure, patient impact, societal impact

## Abstract

**Introduction:**

Translational research is a subfield of the biomedical life sciences that focuses on clinically driven healthcare innovations. The workforce of this subfield, i.e., translational researchers, are diversely specialized and collaborate with a multitude of stakeholders from diverse disciplines in and outside academia in order to navigate the complex path of translating unmet clinical needs into research questions and ultimately into advancements for patient care. Translational researchers have varying responsibilities in the clinical, educational, and research domains requiring them to split their time two- or three-ways. Working between these domains and alongside peers who do not split their time as such, raises questions about the academic reward system used to recognize their performance, which mainly focuses on publication metrics within the research domain. What is unclear is how combining research tasks with tasks in the clinical and/or educational domains effects translational researchers and how they navigate the academic reward system.

**Methods:**

In this exploratory interview study, semi-structured interviews were conducted to gain a deeper understanding of the current academic reward system for translational researchers. Stratified purposeful sampling was used to recruit 14 translational researchers from varying countries, subspecialties, and career stages. The interviews were coded after data collection was complete and arranged into three overarching result categories: intrinsic motivation, extrinsic factors, and ideal academic reward system and advice.

**Results:**

We found that these 14 translational researchers were intrinsically motivated to achieve their translational goals while working in settings where clinical work was reported to take priority over teaching which in turn took priority over time for research. However, it is the latter that was explained to be essential in the academic reward system which currently measures scientific impact largely based on publications metrics.

**Conclusion:**

In this study, translational researchers were asked about their thoughts regarding the current academic reward system. Participants shared possible structural improvements and ideas for specialized support on an individual, institutional, and also international level. Their recommendations focused on acknowledging all aspects of their work and led to the conclusion that traditional quantitative academic reward metrics do not fully align with their translational goals.

## Introduction

**Figure 1:**
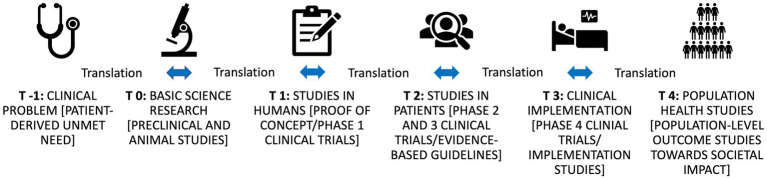
The translational research pipeline.

Research-driven healthcare innovations improve patient care across the entire patient journey, from diagnosis to treatment and from prevention to quality of life (1). These innovations come about through the work of committed professionals in the life sciences field. Translational research is a subfield of the biomedical life sciences that turns observations of unmet patient needs into interventions that improve the health of individuals and the public (2). The term translational is used to describe the iterative process of translating clinical problems from patients into research questions that are then translated again into viable solutions that ultimately impact patients’ lives (1). All research along these lines is part of the translational pipeline, this involves basic research, preclinical research, clinical research, clinical implementation, public health, and patient involvement (Figure 1).^1^

Translational researchers constitute the workforce of translational research. They combine varying levels of responsibilities in the clinical and/or research domains while often also being involved in education. Translational research is highly multidisciplinary with opportunities for specialization into clinical medicine, molecular research, pharmaceutical science, epidemiology, community health, population, and policy science (3). Creating solutions that impact patients requires adequate knowledge, robust methodology, and long-term collaboration across multiple disciplines, involving stakeholders from both academia and outside, who work together to navigate the complex path towards successful translation (3). However, translational researchers must balance multiple roles simultaneously, including coping with unaligned priorities within the clinical and research domains, educational roles, and financial burdens (4). This balancing act is not always maintainable and some translational researchers struggle to manage their often splintered affiliations (4).

Most of the literature on translational research focusses on a subset of translational researchers: physician-scientists. These are translational researchers that often hold an MD as well as a PhD degree and predominantly work in academic settings. Physician-scientists bring a valuable perspective to translational research because they are in direct contact with patients and thus form a link that steers research in the direction of patient relevant outcomes (5). However, according to Hurst et al. (5) the amount of physicians attributing a sizeable portion of their time to research has decreased in the last 40 years (5). In fact, the term “endangered species” has been used since 1979 to describe what was then called “clinical investigator” but is now known as a translational researcher (6). According to Brown et al. (7) the global downward trend in the number of physician-scientists is caused by structural faults within the career progression of translational researchers (7).

Translational researchers have varying responsibilities in the clinical and educational domains. However, their success is mostly judged based on their scientific publication achievements which drives funding and helps further researchers’ careers but does not directly help patients (8). This divergency emerged in the 1970s when rapid technical advancement of molecular biology began to separate clinics from science and sprouted the hypercompetitive scientific environment of biomedical sciences (9). Butler (9) stated that this system holds little incentive to promote translational research as choosing to spend time on the clinical implementation phase of the translational pipeline takes away from doing what the system rewards, namely producing publications (9). Fernandez-Moure (1) went on to link the limited amount of time available for this clinical implementation to employers’ priorities and demands for funding and publication output. They explained that a lack of financial support limits research time for clinicians and that funding demands limit time to implement research findings for researchers (1). This cycle of funding and publication output is even more pronounced for translational researchers because they must balance their multiple domains, therefore, the emphasis on publications creates structural faults within their work settings. This has the potential to drive out translational researchers because they are unable to maintain status quo.

To increase the number of physician-scientists, Brown et al. (7) described multiple initiatives that have been created to help reverse the declining numbers, such as moving away from publication pressure and publication counting (7). Recent discussions in the scientific community about alternative ways of rewarding researchers have addressed the direct link between the number of publications, citations, journal impact factor, and a researcher’s h-index to measure success (10–17). While these initiatives focus on improving some of the imbalance within the career progression of translational researchers, no overall viable solution has been established. The discussions in this area are largely in the form of perspectives, commentaries, and editorials. In a perspective by Moher et al. (10) recommendations from an expert panel reported the misalignment of faculty incentives and rewards with the needs of society (10). The focus of their discussion centered around the reproducibility crisis and suboptimal quality of the publication system. The use of journal impact factor in academic review, promotion, and tenure evaluations was also addressed in a study by McKiernan et al. (11), which raised concerns about its misuse in evaluating the quality and significance of research (11). Moustafa (12), in their commentary, went as far as calling the misuse of the journal impact factor a disaster (12). In 2016, Nature published two items that focused on impact factor as an unfit measurement for clinical impact that encourages quantity over quality and perverts research priorities away from unmet patient needs (13, 14). In their perspective, Casadevall and Fang (15) named the persistent misuse of the impact factor an epidemic mania that afflicts all researchers (15). In an editorial a year later, they wrote that science has always been competitive, however, adverse effects on creativity, resource sharing, and research integrity are now apparent (16). While its creator Eugene Garfield had intended to create an algorithm to ostensibly measure the importance of scientific research, he recognized early on that “like nuclear energy, the impact factor was a mixed blessing” and that “in the wrong hands it might be abused” (17).

Consequences of the culture surrounding the push for publications have also been discussed. In their study Tijdink et al. (18) found that the current research system focused on publication led to counterproductive stress, negative sentiments, and questionable research practices (18). Adding to this, Alberts et al. (19) explained in their perspective that researchers now find themselves in an “unsustainable hyper-competitive environment that is discouraging for prospective researchers” (19). In their study Quan et al. (20) described great monetary advantages for authors who publish in high impact journals replacing the goal of publishing to disseminate knowledge with personal gain, coining the term “publish or impoverish” (20). Trends in publication behavior were also addressed in an editorial by Tshomba and Cavalli (21) and study by Wesel (22) exposing the strive for publications in high-impact journals and the use of citation metrics in an evaluative way (21, 22).

All of these studies and discussions highlight the need for a deeper understanding of the academic reward system, especially in regard to translational researchers, who find themselves juggling responsibilities outside of the research domain but are still held accountable by its reward system. To understand how this publication-focused reward system influences the work of translational researchers, we designed this exploratory interview study consisting of semi-structured interviews with translational researchers from different countries, subspecialties, and at different career stages to help us understand how translational researchers perceive the current academic reward system within their career pathways. In order to answer this question, we investigated the current academic reward system and institutional structures that are in place to reward translational researchers. The aim of this study was to understand what motivates translational researchers to continue their work in an environment that is not conducive to all aspects of their work, in addition to highlighting actionable points of improvement regarding the current academic reward system. Ultimately, we hope this research will contribute to the continued advancement of translational research by understanding the best way for rewarding all aspects of its main workforce.

## Methods

### Study context

In 2017, a European strategic partnership consisting of the University Medical Center Utrecht, University College London, Ghent University, University of Granada, and Nutricia Research BV procured a three-year Erasmus+ KA203 (Strategic Partnerships for Higher Education) grant within the KA2 (Cooperation for Innovation and the Exchange of Good Practices) category for the PATHWAY Project. The aim of the PATHWAY Project was to aid the advancement of translational research by supporting the career pathways of translational researchers. As part of the PATHWAY Project deliverables, this exploratory interview study was designed to gain first-hand insight into the current working experiences of translational researchers. The granting authority performed project reviews and audits to ensure compliance with the grant agreement rules.

### Study participants

To collect a variety of perspectives for this exploratory interview study, a stratified purposeful sampling technique was used to increase the credibility of our research findings and to facilitate comparisons between interviewees. The sampling stratification focused on geographical locations, educational backgrounds, work experiences, and areas of expertise. Fourteen translational researchers were identified by the project’s Principal Investigator (PI) through the consortium’s own network and the networks of their associated partners: the European Network for Children with Arthritis/Pediatric Rheumatology European Society (ENCA/PRES), Eureka Institute, Ljubljana University, and University of Toronto. They were invited via email to participate in our study and all 14 researchers agreed. It must be noted that these participants were not part of the PATHWAY project team.

The interviewees consisted of 12 MD/PhDs and two PhDs (nine male and five female) employed in nine different countries within Europe, the United Kingdom, and North America, representing diverse subspecialties (e.g., pediatrics, rheumatology, neuroscience, psychology, cardiology, pharmacology) and varying years of work experience (e.g., from MD/PhD candidate to 29 years post PhD). Futher information was withheld to protect the anonymity of the study participants, see Table 1 for a breakdown of their characteristics.

**Table 1:** Study participant characteristics.

Interviewee	Female/Male	Employed in	MD/PhD	Years post PhD
1	Male	Europe	MD PhD	22
2	Female	Europe	MD PhD	12
3	Male	Europe	MD PhD	29
4	Male	Europe	MD PhD	8
5	Male	North America	MD PhD candidate	N/A
6	Female	Europe	MD PhD	17
7	Female	United Kingdom	MD PhD	6
8	Male	Europe	MD PhD	19
9	Female	Europe	MD PhD	8
10	Male	North America	PhD	15
11	Male	Europe	MD PhD	10
12	Male	United Kingdom	MD PhD	16
13	Male	Europe	MD PhD	3
14	Female	Europe	PhD	11

### Study design

The 14 participants were invited via email for a 45-min online semi-structured interview with the first author using Zoom video conferencing. The interviews took place in October and November 2020. Before the start of each interview, oral informed consent was obtained for the study and to record the audio of the conversation. To protect the privacy of the participants, participant information was anonymized and given a number for further data processing. Questions asked during the interviews covered the participants’ current responsibilities within the clinical, educational, and research domains. Interviewees were asked to explain how they were able to combine their separate domain tasks in order to further their translational goals. They were also asked to put into context how they were rewarded for their work in each of the three domains (clinical, educational, and research) and to name areas of improvement. A full list of interview questions can be found in Appendix I.

### Data analysis

To preserve the authenticity of the interviews, an intelligent verbatim transcription was done in Microsoft Word eliminating pauses, repetitive wording, and inserting context where needed to ensure more clarity of the interviewees’ answers. The full intelligent verbatim transcripts can be requested. The information from each interview Word file answering the following six interview questions was then copied into Microsoft Excel: (1) What is the definition of a translational researcher in your eyes? (2) What is your personal goal within translational research? (3) Which categories can your work be divided into? (4) Would you need to divide your time differently to optimally achieve your personal goals? (5) What is your advice for early-career translational researchers? (6) What is your advice for policymakers?

Six additional thematic categories were identified in the texts during analysis and highlighted in each Word file before being grouped in Excel: (1) Balancing multiple roles; (2) Current clinical reward system; (3) Current educational reward system; (4) Current research reward system; (5) Areas of improvement; and (6) Financial burdens. After reviewing the Excel file containing the answers of all 14 participants to the six questions and six additional identified themes, all authors agreed that the data should be categorized into: intrinsic motivation, extrinsic factors, and ideal academic reward system and advice.

All coding was done after data collection and sorting was complete. Using descriptive coding, labels were assigned to mark the first subcategory within intrinsic motivation pertaining to what goals the interviewees want to achieve within translational research. This coding method was used to identify the main themes within this subcategory: for themselves, for research, for patients, and beyond research and patients. For the second subcategory on how the interviewees want to achieve their goals, process coding was chosen to help identify specific actions within the data. This led to the themes: connecting and collaborating, generating new knowledge, and clinical development. Descriptive coding was used again in the extrinsic factors category to sort data into clinical, educational, and research domains. This section was re-sorted into the subcategories: scheduling and priorities, reward systems and metrics, and the impact of extrinsic factors on intrinsic motivation. Evaluation coding was then used to decipher positive and negative remarks of the interviewees to assign judgment about the extrinsic factors affecting their current working systems. Data regarding the category ideal academic reward system and advice was coded using both descriptive and process coding to capture the multi-level nature of this final category pertaining to ideal situations and advice on the subcategories: individual level, institutional level, and international level (23).

## Results

The results of this study were divided into three main categories describing what the 14 interviewed translational researchers reported after being asked about various facets of their working lives: intrinsic motivation, illustrating what participants want to achieve within translational research and how; extrinsic factors, descriptions of current working experiences in the clinical, educational, and research domains and how these affect their intrinsic motivation; and ideal academic reward system and advice, possible multi-level adaptations, and advice for early-career translational researchers in addition to policymakers on how to make these adaptations. To improve the readability and clarity of the findings, quotes have been further edited from the intelligent verbatim transcription, omitting unnecessary and repetative sentences and phrases without affecting the meaning and tone of the interviews.

## Intrinsic motivation

To understand how translational researchers perceived the academic reward system within their careers, we first explored the intrinsic motivation of participants and their goals within translational research. Their answers were separated into two subcategories: what goals interviewees want to achieve, and how interviewees want to achieve their goals.

### What goals interviewees want to achieve

The first subcategory was sorted into four themes: for themselves, for research, for patients, and beyond research and patients. Six interviewees stated goals for themselves. Their answers related to feelings of happiness and social responsibility, achieving a sense of purpose and fulfillment, being useful, and being a better clinician:

“The research and teaching are something I do because I like it, just for my motivation. […] The satisfaction is something personal, I feel my career is more brilliant. I feel more satisfaction, and I think in life what you need is to be happy, and if I’m happy doing this I don’t need an external reassurance of what I’m doing. Being useful for the science, that’s the main reason why I do this. […] I think my career is better, […] because I think you are a better clinician if you are also a researcher.” (Participant 11)

Goals for research were mentioned by two interviewees, with one mentioning discovering and gaining knowledge, and another mentioning understanding the impact of a certain condition and how to treat it:

“My overall goal is to better understand the real-world impact of [condition x], what those impacts mean physiologically and what we can do about them. […] I’m less disease focused and more health focused.” (Participant 10)

Goals for patients, stated by six interviewees, included achieving the application of new diagnostic tests and therapies in addition to improving patient outcomes and quality of life:

“My personal goal within translational medicine is to use the work that I do to create better quality of life for patients. […] That’s a niche that I think has gone very much underappreciated, and so that’s where I found my role.” (Participant 5)

Goals beyond research and patients were mentioned by five interviewees. Answers revolved around the broader sense of creating impact, from common knowledge to clinical practice, and beyond:

“The idea that my science could impact either common knowledge or clinical practice is satisfying, but that is ephemeral, because whether or not it will actually [have impact] and how, and how much and to what extent […] is something I’ll see years down the line.” (Participant 13)

### How interviewees want to achieve their goals

The second subcategory was sorted into three themes: connecting and collaborating, generating new knowledge, and clinical development. For connecting and collaborating, four interviewees described activities related to underpinning clinical studies with basic science and building bridges between people and fields to progress research:

“[To achieve my goals] I feel like the majority of my time is spent more on the population community side, but a lot of my brain time is spent in between a lot of these [domains]: helping to bring community research into a more translational perspective, and helping to bring basic research into a more translational perspective, and helping to get clinicians to think a little more in both directions.” (Participant 10)

“By making effective and fulfilling or rewarding collaborations, by developing myself as a scientist, and by making inventions, or by actually making progress in the field.” (Participant 1)

For generation of new knowledge, five interviewees mentioned activities within research. Answers varied from understanding the pathophysiology of a disease, predicting disease courses, detecting and validating novel disease markers, and developing research models:

“The functional validation of the novel genetic variants will not only improve the diagnosis in this patient but will also improve the knowledge on the underlying pathogenesis, and […] with this improvement of knowledge on underlying pathogenesis, this will improve or, let's say, enhance novel therapeutic possibilities.” (Participant 9)

“If you look at the translational aspect, my goal would be to develop a [model] for [condition x], so that we can do studies in it to really improve this outcome.” (Participant 6)

For clinical development, five interviewees described activities supporting patients. These included the improvement and development of diagnostic assays, as well as early disease detection tools:

“We are developing early risk stratification tools for early detection and with that we hope to, in the sense of benefiting patients, have an impact on the way these programs are structured, with the ultimate aim of providing a cost return to larger health care systems because it would be easier, in the end cheaper, to catch patients early rather than to see patients at a later stage of disease.” (Participant 12)

## Extrinsic factors

After discussing their goals, participants were asked about external influences that affect their work. They provided statements about their current working experiences which were divided into three subcategories identified within the overarching clinical, educational, and research domains: scheduling and priorities, reward systems and metrics, and the impact of extrinsic factors on intrinsic motivation.

### Scheduling and priorities

Within scheduling and priorities, clinical and educational tasks were described by eight interviewees as taking priority over time for research. Answers varied from having to earn research time while fulfilling clinical and educational duties, to allocating time outside of work hours to review research papers, secure research funding, as well as sacrificing one’s own research time to help more junior researchers:

“My clinical work is getting very demanding, and I usually have to use my free time at home. I don’t deny that when the children go to bed, I start with the computer to review the papers, to see the databases, and that kind of thing.” (Participant 11)

“For me it's difficult to say ‘OK, now I will focus on my research work.’ I still try to have some protected time, but this is usually protected time at home not at work. At work my research time is more focused on helping younger colleagues in their research. I'm much more organized with reviewing other research than my own research because I try to be responsible to other colleagues, I think that I have to be in this respect, consistent […]. So, I try to adapt my balance to incoming duties, clinical, research, educational. But it's a constant struggle.” (Participant 8)

Six interviewees explained scheduling and priorities to also be challenging. Answers varied from misunderstanding between different work cultures and ideological differences from peers and seniors about their different roles outside of clinical work, managing the administration of their combined roles, to meeting the expectations of employers with different priorities:

“I'm always trying to balance between the time I would dedicate to science, but also the time I have to advance in [my clinical subspecialty] because […] I have sick kids near me, and I have to do my best to help them as best as I can when I admit them. So yeah, I'm just trying to swim. I would say that the real conflictions are that my surroundings, like in the hospital, they don't have a clear view of a translational scientist and they don't understand that somebody would like to do science. There is absolutely no education in this way.” (Participant 4)

“When I'm in my clinical role, there's always things like meetings that certain researchers can only do obviously on the day that I've got a clinical thing. So, it's trying to fit those things in without upsetting the clinical team and without people thinking that I'm reducing my responsibilities and am not interested. And then in the other direction, when I'm on a research day, I might get the secretary from the clinical saying this patient wants to get a hold of you, or have you seen that letter, can you sign it off, or can you come and help us with this clinic because so and so is off. So, I can get pulled in the other direction as well.” (Participant 7)

### Reward systems and metrics

All participants were asked to describe reward systems and metrics, i.e., systems or standards of measurement regarding evaluations that they were aware of for their work within the clinical, educational, and research domains. Regarding the clinical domain, one interviewee stated that there was no reward system for clinical work, while another explained that metrics for clinical work existed, however, it was unclear if they were used for evaluations:

“I mean there are metrics for my work in the clinic, meaning how many patients opt to be seen by me specifically, how long a waiting list I have, and I get the sense that patients are pleased with my work because my outpatient clinic is constantly full. […] I guess metrics would be available for that if I asked my hospital administration, it never occurred to me to do so because I get a very immediate reward from patients […] I don't need a metric for that. Also, I am not being evaluated by my hospital based on these metrics or they probably do, but they’ve kept it to themselves so far.” (Participant 13)

Regarding tasks in the educational domain, four interviewees said that there was almost no known reward system. Participants mentioned it was just part of their job, and while valued, it was not seen as an important aspect of their job or evaluated as such. They said that its impact was difficult to compare to publication counting but that some institutions weigh educational activates as part of academic performance:

“Publications are simple, you have them, or you don’t. Education is very vague. You could hold an educational event with, let's say 100 people and then you could hold another separate educational event with 1,000 people, but the impact of the first educational event, even though it had less people, could be greater. […] Because of that fluidity of education work, or the fluidity of even patient advocacy work, it's exceedingly difficult to put a grade on it, or a way to compare it to other forms of academic work. I think that's one of the biggest challenges. How do we quantify something that in its very nature is very qualitative? I would not be surprised if that's the biggest reason why institutions have had a hard time moving away from this publication merit system and being able to give merit and credit to other forms of qualitative work that psychologically are very important and do great things for society and for patients. […] Thankfully there are institutions that have a credit system where educational activities are weighted as well as looking at your academic performance. If you were to run an educational activity, or a patient engagement activity, there are some institutions that are beginning to look at these things. But in other places, where it's old-fashioned and all they look at is your publication record, it's very challenging to allocate time to things that you feel are more impactful when they're not leading to a publication that your boss thinks has more impact.” (Participant 5)

The reward system and metrics within the research domain were discussed with all 14 interviewees. Their answers have been organized into five subthemes: publications, publication pressure, combining domains, financial situations, and overarching remarks.

Regarding publications, answers varied from the use of publications to inform colleagues about research findings, obtaining funding and future collaborations, to job security. Participants also mentioned that publishing in journals with higher impact factors did not necessarily mean higher impact in their fields. Publications were explained to be the main measurement for gauging success, however, it was also stressed that this was neither reliable, transparent, or valid:

“As a postdoc you need to publish, otherwise you cannot ask for money if you have no manuscript or some kind of proof that you're doing good work or have good ideas. […] If you don't publish manuscripts in high impact journals, the chances are small that you get a scholarship or a PhD student, or money to get your project going and that's a shame. […] In academia you have to basically fight for your own money. […] After your thirties and after you did your PhD and postdoc-ed etc., you might want to start a family. But it's very difficult, at least in my opinion, I found it pretty difficult to start a family without knowing whether I have a job the next year. Because most projects were for two years, maybe four if you had a lot of luck, and it was just the uncertainty that I hated.” (Participant 14)

“Publications are a terrible measurement of success. I would argue that they're neither reliable nor valid. They're just objective, and so, if we were going to use any of these metrics in our experiments as a measure of an outcome, we would never be able to justify it. What does number of publications measure? First of all is it reliable? Well, it's not reliable, because every single field, subfield and sub subfield, has different journals that they publish in, with different types of impact factors, with different scopes. I feel a lot of it [counting publications] is pseudointellectual handwaving nonsense. […] It's not intellectual because it's actually a poor metric, and if you ask anybody, they all know it's a poor metric. You're comparing numerators without adjusting for denominators, which is what the impact factor was supposed to solve. But even across fields, impact factors mean different things. […] If number of papers becomes important, it shouldn't necessarily matter where the papers are [published]. […] My fourth most cited paper is in a journal that isn't even in PubMed by default and it's not in a journal that anyone would find remotely impressive, but it's quite impactful.” (Participant 10)

“Sometimes it’s not clear how you are able to publish in one journal or another; you have a name, or you don't have a name. I've seen very good works that have not been accepted, the group is not very important in the world, and then you see very weak papers from very important groups, and that's something that could be better. […] Ultimately, I prefer a researcher who does just one work in one year but very high quality, than the one that did ten papers but are not really useful, so that's the problem of this system.” (Participant 11)

Regarding the second subtheme publication pressure, external pressure to fulfil faculty requirements, along with internal pressure to be seen as being productive were mentioned. One interviewee stated that at their institution, publications were not the main focus of an academic career but that a person’s network played an important role. Two participants described feeling pressure to publish during the beginning of their career, while others reported that publication pressure created constructive competition amongst their colleagues. Publication pressure was also mentioned as potentially creating a detrimental hierarchal system for researchers, which has now lead to reevaluating the use of publication metrics at some universities:

“I didn't receive constant pressure by the institution, but I know that unfortunately there's a linear correlation between how many papers I publish and my career advancements. I want to stress that it's a quantitative correlation, not a qualitative one.” (Participant 13)

“The pressure to publish is one of these metrics that people judge you by and this is the reality of the world we have to work in. There is internal pressure because the idea is that if you're not publishing, you must not be productive […] and there's external pressure because […] you're expected to have a certain minimum number of publications of varying impact. […] Once I get to the point where I'm a fully appointed professor or assistant professor, the metric is how much you’re publishing in a year, and that's how you keep your job, and that's how you get promoted, so it's a harsh reality of the world that we live in.” (Participant 5)

“It [publication pressure] comes from a pressure to be promoted, but there's peer pressure as well, a sort of pecking order within the institute, who's better, who's best? I know that the fellowship that I'm on […] will have to be renewed, and I need to make sure that I have enough publications on the bill to make that a credible proposal, because I will have to put in a new proposal for the next five years with a budget and I know that reviewers immediately go to your publication page to see what your output has been over the last five years.” (Participant 12)

The third subtheme addressed the challenges of combining responsibilities across multiple domains while being evaluated on the same criteria as non-translational colleagues. Answers varied from difficulties meeting standards and goals to being at a disadvantage when competing for research grants with non-translational colleagues who have more time for research:

“Trying to do everything well is difficult. So, trying to meet all your research goals when you’ve got all this other stuff going on, is difficult, so you might set yourself this list of tasks and only get halfway through and then before you know it, you're back on a clinical day and then you just can't do it. Or similarly, with the clinical side of things, comparing yourself to other clinical trainees who aren't doing any research, who are just doing clinical all the time, they will be much better clinically. […] It's just quite difficult to do both of them really well, and difficult to stay up to date with all the clinical stuff, as much as someone who's doing that all the time, in terms of continuity. So, I might see a patient when I'm doing clinical work and then the next week I'll be doing research and might not find out what happened to them. […] I think it's this constant push and pull in both directions and feeling like you're not doing either of them to the standard that you'd like to. Not feeling like you're completely failing, but feeling like this isn't satisfactory to me, the level of what I've done in this or in that. […] Also, as a clinical academic we all get allocated medical students for different projects. […] So, I'm getting all these students, but with far less time than the people who are full-time academics.” (Participant 7)

“Let's say that if I would have 100% time for research, then of course I would have more time to write grants. […] The competition is not always fair because I don’t have 100% time for research and to be as innovative as other people who do. […] In the past, I may not have complained about getting grants, but I was really afraid in the beginning because I don't have 1% time to do research, while I was still needed to apply for the same grants as other people who are doing 100% research.” (Participant 9)

The fourth subtheme addressed the financial situations translational researchers face when performing their jobs. Participants mentioned not receiving any additional salary as a PI, that their research salary had to come through grants, and that they often have to make financial sacrifices in order to continue their work:

“In terms of research, I'm not receiving any additional money. […] As a scientific director, I'm not receiving any supplementary salary, and this is not fair. […] The grants here are for hiring PhDs and paying their salaries, or for getting consumables and so on. So, it’s different to other European countries where PIs also receives additional funding. […] My basic salary now is very low, […] and the only institution that is paying me is the university. So, I don't receive a second part or a supplement as a researcher. I don't receive a supplement as a clinician. […] I always complain because here the money goes in a very scattered way, and [they] give small amounts of money to each research group […] [which makes it] difficult [to publish] in high [impact] journals, […] so [to achieve] very good publications, with a small amount of money, and because of this scatter, you are limited, and you cannot go beyond. […] How can I compete with people that have these possibilities. This is a major problem for us.” (Participant 3)

“That's what translation really is all about. It's going into this area that's completely unknown. We don't know how to measure it. But there's this feeling in our hearts that it's the right thing to do, and we have to go for it, and for a lot of translational researchers, what that ends up becoming is the realization that you need to take a pay cut somewhere to be able to do what you love and what you think is important. It's much more lucrative from a salary perspective to just do 100% clinical work. You can live lavishly. You can make tons of money. You won't have to worry about job security. But it's just a loop and you'll be stuck in that loop, and you won't be able to change the status quo.” (Participant 5)

Finally, the current overarching reward system of the research domain was discussed. One interviewee mentioned that the only reward system they knew was in research and that this system was not working properly. Another participant said they had no knowledge of formal rewards in the research domain but that informal rewards included respect, freedom, opportunities for collaboration, and how their work impacts people:

“For the research work there is this rewarding system of publications and impact, and the system of the grants that you receive or manage but that is […] not really doing what it should do. It's not rewarding what it should in my opinion. […] But it is something, so people tend to use it […] but there is virtually no rewarding system for the other fields.” (Participant 1)

“The rewards are respect in the field. Rewards are the freedom to ask the questions that I want to ask and to do the projects that I find interesting and fun to do. The rewards are, you know, respect from peers. Rewards are opportunities to collaborate with fun people and do fun things. Those are the rewards, and another important reward is feeling like the work that I'm doing is making an impact on actual people, and is interesting to people. I mean that's a reward in and of itself.” (Participant 10)

### The impact of extrinsic factors on intrinsic motivation

The final result category explored how the current working experiences of translational researchers influenced their work and how these extrinsic factors affected their intrinsic motivation. Three interviewees described clinical work to be intrinsically rewarding and that no further external rewards were needed. Two of the three interviewees who gave this response also said the same about teaching. However, one interviewee specifically mentioned they were not happy with the lack of recognition about their translational work:

“The inner reward is the only kind of reward I can get. There is no recognition. There is no salary. When I speak about what I do at conferences, that's also rewarding, when I spread it [the research]. I like to talk to students about it [the research]. I think that the only chance to change something is by intervening with new generations. We don't have the infrastructure [referring to their country]. […] If you're applying for a grant, you have all the basic principles like in every other European country. But in practice this doesn't work. They ask about the amount of time, your head of institution even signs that you're only allowed to work this certain amount of time in science, but nobody actually follows this. They don't care about this […] other colleagues don't understand this. They don't like it. They don't get it. Why are you doing this? They don't see the reason.” (Participant 4)

The impact of extrinsic factors on intrinsic motivation within research was described by four interviewees. Answers ranged from feeling respected and freedom in their work, to having close patient relationships and creating patient impact. Publications were mentioned as not intrinsically motivating and that years of work culminating into a publication had a protracted sense of fulfillment:

“I really don't see publications as a reward at this point. Of course, you need them. But if I were to say that I extract my emotional satisfaction from publications that will not be true. I'm actually satisfied when I submit a paper and then my emotional attachment to that paper ceases to exist, and that's good because oftentimes you get dismembered by some reviewer, so I wouldn't say that publications are my reward.” (Participant 13)

## Ideal academic reward system and advice

Following the discussions on intrinsic motivation and extrinsic factors, interviewees were asked to provide statements on what they felt would be the ideal academic reward system regarding their translational work and advice to early-career translational researchers and policymakers. The results were divided into three subcategories: individual level, institutional level, and international level.

### Individual level

All interviewees gave advice on the individual level. Answers included finding and following what intrinsically motivated them and being a good advocate for the translational field, as well as being dedicated, well-organized, and having good time management skills. Participants also mentioned that early-career researchers should find the right environment to develop and grow, and to find a peer mentor just one step ahead of them. Regarding translational work, interviewees mentioned that leading the change sometimes meant taking criticism and to show active efforts to inform the community about research to help foster accountability, transparency, and education:

“If you don't have a sense of internal gratification or internal drive, you are going to get burnt out and it doesn't matter how many grants you get, it doesn't matter how many publications you get, if you don't maintain and foster that internal sense of why you're doing this, the external rewards will not be enough. […] Thus far, the best way that I have dealt with this conflict [working as a translational researcher] is by being a very good advocate for the work that we do, and showing that through academic means, through personal means, through collegiality with colleagues, how important and fundamental the work [of a translational researcher is] and why it is necessary. So, you begin from the ground up to change the minds and the ideologies of those people around you, so that they recognize how important these things are and that [translational researchers] are really working within a niche that people have forgotten about.” (Participant 5)

“Life in research is very hard, you need to be a very dedicated person and you have to make sacrifices, sometimes personal sacrifices. […] I consider myself a very well-organized person in terms that if you’re trying to do four jobs, which is what I have right now, you need to be very organized in terms of schedule.” (Participant 3)

I would say, it’s often said go and find a mentor, and the advice then tends to imply, go and find a professor who’s achieved that goal that you want to reach. Actually, there should be a greater emphasis on finding peers who are maybe just one small step ahead of you.” (Participant 12)

### Institutional level

When asked to give advice to policymakers, 12 interviewees discussed what institutions could do to help translational researchers. Equally rewarding work in all three (clinical, educational, and research) domains was suggested, as well as having engaged superiors who understood their translational goals. It was mentioned that institutions could also help translational researchers by supporting continuous employment while they navigate their different roles and by combining evaluations for clinics and research to avoid duplication. One interviewee explained the need for a culture shift to a more qualitative reward system, while another said that metrics such as number of publications could be involved in evaluations, however, not solely, and that context should be thoughtfully considered. Lastly, one interviewee mentioned that policymakers should look at research more as a long-term investment in human capital and should invest in supporting researchers to build long-lasting projects that result in clinical changes:

“Dedicated clinicians, dedicated researchers, dedicated educators, […] all being rewarded in a similar way. […] For a good academic hospital, you need all three categories well represented. […] Some people will value research higher than clinics and some other will value clinics higher than education, but for me I’m very unfond of all the comparison things that we’re doing now. […] You can never completely compare the different specialties. […] I think if we had the feeling that we want to be a top hospital on all three domains and we’re happy with everyone who’s contributing to that, that would be the best reward to me. […] Put people in places where they’re best and let them do what they’re really good at and what they really want to do. […] that’s a principle that you see coming around, people who are really good at something and then they become the head of the department and they have to do a lot of management things and they’re not specifically good at that. So, I’d like to invest in the people who are really good at what they’re doing in remaining there and then they don’t need to be the boss.” (Participant 2)

“I think places that are being more thoughtful are considering context. I think they're not removing the metrics, but they're saying […], ‘What is the quality of those publications?’ So that's where you can get into things like citation counts, but even with citations you have to look at that in context, because some fields cite heavily, and some fields cite sparsely. I think places are becoming increasingly flexible, and to be honest, I think the innovation is happening not in the places of privilege. I think that the institutions who have no incentive to change, are not changing. […] There're many institutions that [make you] feel like it's a privilege to work there and to associate with their name, and don't have the motivation to evaluate themselves because they don't really care, they don't care that these measures are somewhat arbitrary, because they're good enough and they're hard to reach, and then being hard to reach is itself a test that they’re willing to put and place on people, even if the metrics are stupid and invalid and unreliable, at least they're difficult and then they can claim exclusivity. But I think honestly, I think that is diminishing, at least from what I've seen.” (Participant 10)

“If they [policymakers] would consider not looking at money or impact points but also at science that is a long-term investment, a long-term strategy focused on implementing therapies or regiments that really make a difference. Then you can start to look at your researchers, the ones that actually have to make these changes, as people that you want to nurture. So, you don't want to only calculate the money they bring in or the impact points they make but also the collaborations they can build, the research lines they can build, that will have a long-lasting stream of inventions, changes, implementations. The impact points and the money is short-term, and the long-term is actually the changes that this research will make. So, they can focus on research lines that actually will make differences. So, it's not the topic of a research line it is also the fact that this line needs to result in clinical implementations. So, we have a lot of research lines or long-lasting projects, this is not new, but the question is whether this results in clinical changes that is not asked so often, I think.” (Participant 1)

### International level

All 14 interviewees suggested improvements on an international level. Recommendations related to building bridges and collaborations that help the translational field grow and that focuses on creating patient impact. Two participants discussed the need for new ways of measuring scientific impact and the faults of a fully objective system. While two other interviewees mentioned removing financial pressures from researchers, especially early-career researcher and those wanting to start families, in addition to asking for different contractual rules from the government to be able to keep longer-term academic positions. Additionally, one participant recommended translational research be recognized as an independent career, while another purposed establishing an educational path for translational research, which included core criteria that institutions had to respect. These core criteria, which were described by another interviewee, should contain clear rules for fair competition and equal opportunities between organizations geographically, in addition to being aware of the favoritism towards more famous institutions. Finally, it was suggested by one participant to included more and different stakeholders in the policymaking process, to reflect the diversity of the population:

“It's really disappointing that people tell you, ‘You do great work, you have great ideas, we just don't have the money.’ You’d rather hear, ‘You know what, let’s part ways because we don't agree, your ideas are not the ideas we want to follow’ or whatever. ‘No, your ideas are good, it's just we don't have the money and our government tells us that we can only renew your contract once’ and that's it. Of course, there are ways around that sometimes. […] But after a while, sometimes you have to disappoint people and they leave your network or do something else while it would have been easier if people could just have different contracts.” (Participant 14)

“There needs to be more funding available for early-career researchers to get little grants to build up towards bigger grants. […] It's important that there’re things that don't disadvantage women, so having grants specifically for women who have come back from maternity leave and are already on the back foot and need a bit of money to buy out someone’s time to help them. […] There are other things that can be done around childcare and conference days, maybe a creche at these conference days. If you're getting a bursary to go to a conference or something like that, could there be a child care bursary? […] There's a lot of things that could be done that aren't done to support women, particularly to be able to do everything they want to do.” (Participant 7)

“To the policymakers, I think that they have to recognize the figure of clinician researchers as an independent career. I mean, at the hospital, you need to have full-time clinicians, but also the number of clinician researchers that we have right now, is very small; less than 5%, and these type of people are people that should be leading the research inside the hospital.” (Participant 3)

## Discussion

This exploratory interview study, consisting of semi-structured interviews with 14 translational researchers from different countries, subspecialties, and at different career stages, aimed to provide real-life accounts of the current working experiences of translational researchers and to gather suggestions for an ideal academic reward system that considers all facets of their work. Our study showed that this group of translational researchers is intrinsically motivated to achieve their translational goals. In their current work settings, clinical work was reported to take priority over teaching, which in turn took priority over time for research. However, dedicated research time was explained as essential for satisfying the current academic reward system that measures scientific impact and the awarding of grants largely based on research metrics such as publications, citations, journal impact factors, and h-indexes. The translational researchers we interviewed suggested that for their ideal academic reward system, both a top-down and bottom-up cultural shift is required to allow for more qualitative performance measurements within institutional structures and facilitate understanding between them and their non-translational colleagues.

When looking more closely at the results, one finding that stood out was that while the current reward systems within the clinical, educational, and research domains were reported as not being geared towards translational researchers, this did not prevent them from meeting their translational goals. Time commitment beyond working hours and perseverance to combine domains, even when employers’ demands would not allow it, were reported as necessary for translational researchers in their current work settings. What appears to keep them in this line of work is their strong intrinsic motivation, connected to long-term, domain-overarching goals, and feelings of happiness that come from working towards some form of societal impact. Clay et al. (24), in a perspective on translational medicine training, recognized that identifying and acknowledging one’s own motivations was required to achieve effective training. However, they did not discuss the impact of external factors on intrinsic motivation which was a focal point in our interview discussions (24).

**Figure 2:**
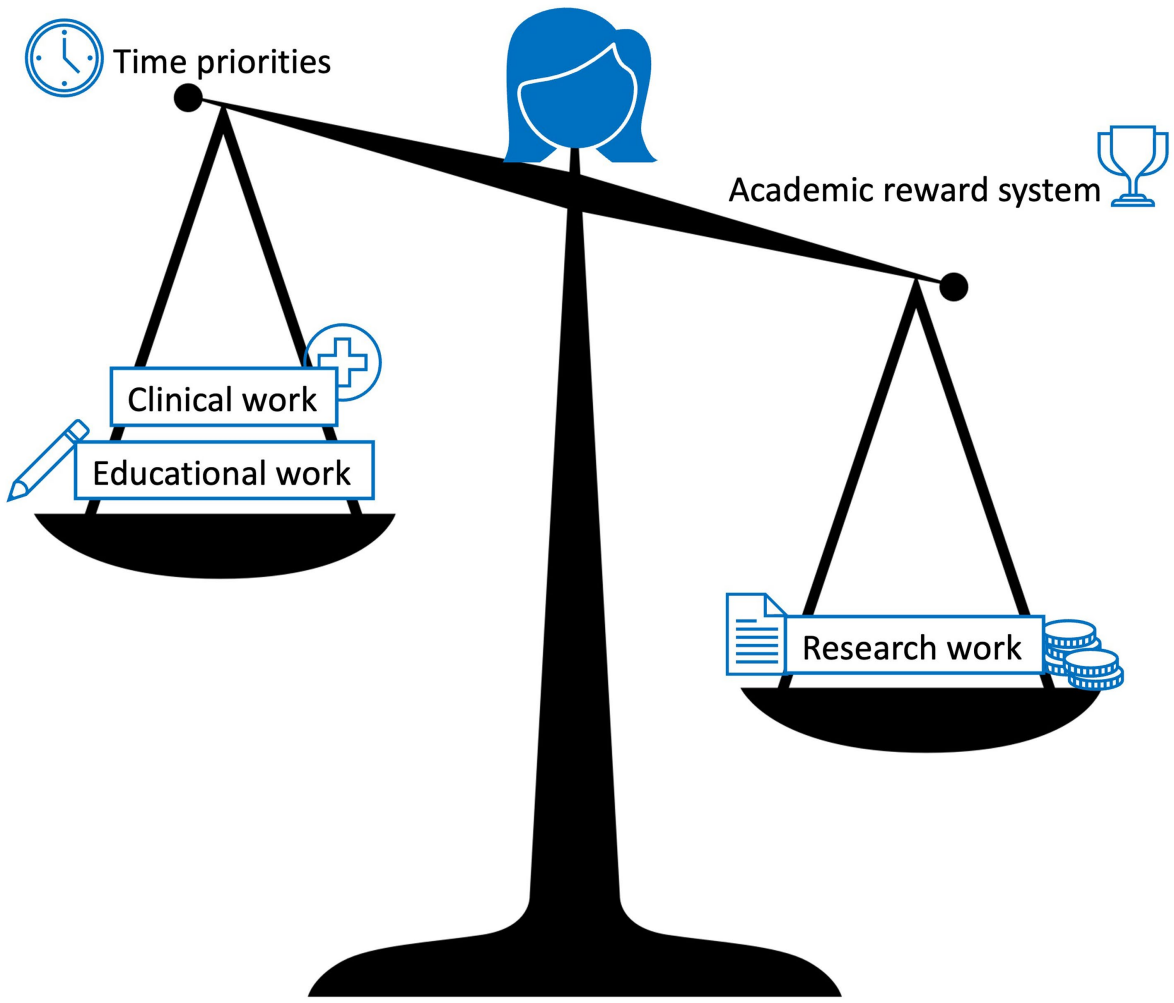
The imbalance of time priorities and the academic reward system for translational researchers. Clinical work takes priority over educational work which in turn takes priority over time for research work, while research work is heavily weighted in the current academic reward system.

External factors, namely the current reward system within the research domain revolving around publication metrics, was proposed as being the main currency of evaluations and the attainment of grants. Reward systems for clinical and educational roles were reported to be less obvious. When time is factored into this equation, translational researchers, who spend time outside the research domain, reported being at a disadvantage (Figure 2). This disadvantage was also said to be apparent when considering how the distribution of time affects translational researchers financially. Several interviewees mentioned having to forgo income to perform their translational tasks. They explained that it could be more lucrative to spend more time in clinics or to have more dedicated research time to secure research grants. To attain their translational goals however, they reported having to satisfy the current reward system within research. This finding aligns with the biomedical literature which has highlighted the negative effects of publication pressure on researchers such as their struggle for dedicated research time, burnout, and scientific misconduct (19, 25–27). This literature also addresses the misuse of the journal impact factor as explained by some of our participants and additionally points out potential biases of the peer review system (28, 29).

Not all translational researchers formally work within the three domains. Two out of the 14 interviewees were not medical doctors, however, they held responsibilities in the clinical domain and their work was closely connected to patient relevant outcomes. This illustrates the variety of roles that translational researchers can hold, and that one solution will not fit all. Rubio et al. (30) agreed that because translational research is not clearly defined, developers of translational research programs struggle to set program objectives, define the knowledge and skills that must be attained, and assess when program objectives and competency requirements have been met (30).

When asked about areas of improvement within the academic reward system, the majority of the interview participants focused on the research reward system, while the reward systems within the clinical and educational domains were addressed less. It was explained by the participants that reward systems in these two domains are less obvious and experienced as more intrinsically rewarding. All interviewees were asked to share ideas on how their current working experiences could be improved and to provide advice to early-career translational researchers. None of the participants advised them to try and change the current academic reward system. The advice, they did share, focused on how to be successful within the current system. Interviewees did, however, provide actionable advice for policymakers, suggesting that performance measurements should take into consideration all tasks of a translational researcher and not just the research domain, which would require a clearer definition of what a translational researcher is and does. To address this need, participants suggested specialized training programs for translational researchers that help create sustainable career pathways with metrics that reward work across all their domains. The advice of our participants concurs with other recommendations that suggest multi-level adaptations for the research system and reorganization to better support translational researchers (31, 32). Further literature in this area also suggests using different measurements of impact, moving away from classic bibliometrics in academia and towards measurements of impact on society and legislation (33, 34). In addition, in 2016, Elsevier launched CiteScore as a rival to the impact factor in assessing the quality of academic journals (35) and other suggestions have been made to counter the traditional use of citation metrics and h-indexes (36, 37).

## Limitations

We used a stratified purposeful sampling technique to select 14 translational researchers. They were from varying countries, subspecialties, and career stages, and identified as being an accurate representative sample to understand how translational researchers perceive the current academic reward system within their career pathways. Extrapolating the results from our sample to the global population of translational researchers must be done with care. These findings provide empirical evidence of the real-life working experiences of these specific participants. Nevertheless, unless otherwise stated, the interviewees’ answers overall aligned with one another, making the information potentially more generalizable. Additionally, all participants came from the network of the PATHWAY project’s PI, and have all been able to navigate the complex work settings they operate in. Future research including translational researchers that have left this subfield would offer additional insights on the sustainability of this career pathway, however, locating them could prove difficult.

## Conclusion

The aim of this study was to better understand what motivates translational researchers to continue their work in an environment that is not conducive to all aspects of their job, and to seek advice on points of improvement within the current academic reward system. Participants provided several suggestions for specialized support on an individual, institutional, and international level. A top academic institution should acknowledge and support different employee tracks, allowing individuals to customize their focus by choosing from various combinations of clinical work, educational involvement, and research. Translational research should focus on healthcare innovations based on patient and population needs, rather than publication metrics. The main finding of this study is that there are currently limited reward systems in place that acknowledge all aspects of the specialized work of translational researchers. However, these translational researchers remain intrinsically motivated to achieve their translational goals. Our findings confirm what previous studies have highlighted, that the work of translational researchers is challenging and that traditional quantitative research reward metrics do not fully align with their translational goals or fully encompass all aspects of their work.

## Data availability statement

The raw data supporting the conclusions of this article will be made available by the authors, without undue reservation.

## Ethics statement

Ethical review and approval was not required for the study on human participants in accordance with the local legislation and institutional requirements. The participants provided their written informed consent to participate in this study. Written informed consent was obtained from the individual(s) for the publication of any potentially identifiable images or data included in this article.

## Author contributions

FK conducted the interviews and authored the manuscript, with CF as substantive editor and proofreader. BP recruited the study participants and together with HR reviewed the final manuscript. All authors contributed to the article and approved the submitted version.

## Funding

FK and BP were supported by EU Erasmus+ grant 2017-1-NL01-KA203-035211.

## Conflict of interest

The authors declare that the research was conducted in the absence of any commercial or financial relationships that could be construed as a potential conflict of interest.

## Publisher’s note

All claims expressed in this article are solely those of the authors and do not necessarily represent those of their affiliated organizations, or those of the publisher, the editors and the reviewers. Any product that may be evaluated in this article, or claim that may be made by its manufacturer, is not guaranteed or endorsed by the publisher.

## Footnotes

^1^The information in this figure is based on Fernandez-Moure (1).

## Appendix I

Semi-structured interview questions:

1. What is the definition of a translational researcher in your eyes?
2. What is your personal goal within translational research?
3. Which categories can your work be divided into?
4. How is your time currently divided between these categories? In percentages?
  - Did this evolve over time?
  - Would you need to divide your time differently to optimally achieve your personal goal?
5. Do you now or have you ever experienced conflicting interests between categories?
  - If so, describe them? How did you overcome them? Are there still conflicts?
  - Why do you think there are conflicting interests? Because of current reward systems?
  - What would be an ideal reward system per category? Do you feel pressure to publish?
  - What considerations do you make between work tasks? Do you agree with the priorities?
6. What is your advice for:
  - Early-career translational researchers.
  - Policymakers.

## References

[1] Fernandez-Moure JS. Lost in translation: the gap in scientific advancements and clinical application. Front Bioeng Biotechnol. (2016) 4 . doi: 10.3389/fbioe.2016.00043 PMC4891347 27376058

[2] National Center for Advancing Translational Sciences (NCATS), National Institutes of Health, U.S. Department of Health & human services. Translational science Spectrum [internet] (2021) [cited 2023 Apr 10]. Available at: https://ncats.nih.gov/translation/spectrum.

[3] Edelman ER LaMarco K. Clinician-investigators as translational bioscientists: shaping a seamless identity. Sci Transl Med. (2012) 4:135fs14. doi: 10.1126/scitranslmed.3004109, PMID: 22623734 PMC4624334

[4] DeLuca GC Ovseiko PV Buchan AM. Personalized medical education: reappraising clinician-scientist training. Sci Transl Med. (2016) 8:321fs2. doi: 10.1126/scitranslmed.aad0689, PMID: 26764155

[5] Hurst JH Barrett KJ Kelly MS Staples BB McGann KA Cunningham CK. Cultivating research skills during clinical training to promote pediatric-scientist development. Pediatrics. (2019) 144:e20190745. doi: 10.1542/peds.2019-0745, PMID: 31363070 PMC6855830

[6] Wyngaarden JB. The clinical investigator as an endangered species. N Engl J Med. (1979) 301:1254–9. doi: 10.1056/NEJM197912063012303, PMID: 503128

[7] Brown AM Chipps TM Gebretsadik T Ware LB Islam JY Finck LR. Training the next generation of physician researchers—Vanderbilt medical scholars program. BMC Med Educ. (2018) 18:5. doi: 10.1186/s12909-017-1103-0, PMID: 29301521 PMC5753449

[8] Roberts SF Fischhoff MA Sakowski SA Feldman EL. Perspective: transforming science into medicine: how clinician-scientists can build bridges across Research’s valley of death. Acad Med. (2012) 87:266–70. doi: 10.1097/ACM.0b013e3182446fa3 22373616

[9] Butler D. Translational Research: Crossing the Valley of Death. Vol. 453, Nature. Nature Publishing Group (2008). 840–842. Available at: Available at: https://doi.org/10.1038/453840a 10.1038/453840a 18548043

[10] Moher D Naudet F Cristea IA Miedema F Ioannidis JPA Goodman SN. Assessing scientists for hiring, promotion, and tenure. PLoS Biol. (2018) 16:e2004089. doi: 10.1371/journal.pbio.2004089, PMID: 29596415 PMC5892914

[11] McKiernan EC Schimanski LA Muñoz Nieves C Matthias L Niles MT Alperin JP. Use of the journal impact factor in academic review, promotion, and tenure evaluations. elife. (2019) 8:8. doi: 10.7554/eLife.47338 PMC6668985 31364991

[12] Moustafa K. The disaster of the impact factor. Sci Eng Ethics. (2015) 21:139–42. doi: 10.1007/s11948-014-9517-0 24469472

[13] Callaway E. Publishing elite turns against impact factor. Nature. (2016) 535:210–1. doi: 10.1038/nature.2016.20224, PMID: 27411614

[14] Benedictus R Miedema F. Fewer numbers, better science—fix incentives to fix science. Nature. (2016) 538(Comment Redefine Excellence:453–5. doi: 10.1038/538453a 27786219

[15] Casadevall A Fang FC. Causes for the persistence of impact factor mania. mBio. (2014) 5:e00064–14. doi: 10.1128/mBio.00064-14, PMID: 24643863 PMC3967521

[16] Fang FC Casadevall A. Competitive science: is competition ruining science? Infect Immun. (2015) 83:1229–33. doi: 10.1128/IAI.02939-14, PMID: 25605760 PMC4363426

[17] Garfield E. Journal impact factor: a brief review. CMAJ. (1999) 161:979–80. PMID: 10551195 PMC1230709

[18] Tijdink JK Schipper K Bouter LM Pont PM De Jonge J Smulders YM. How do scientists perceive the current publication culture? A qualitative focus group interview study among Dutch biomedical researchers. BMJ Open. (2016) 6:e008681. doi: 10.1136/bmjopen-2015-008681, PMID: 26888726 PMC4762115

[19] Alberts B Kirschner MW Tilghman S Varmus H. Rescuing US biomedical research from its systemic flaws. Proc Natl Acad Sci. (2014) 111:5773–7. doi: 10.1073/pnas.1404402111, PMID: 24733905 PMC4000813

[20] Quan W Chen B Shu F. Publish or impoverish: an investigation of the monetary reward system of science in China (1999-2016). Aslib J Inf Manag. (2017) 69:486–502. doi: 10.1108/AJIM-01-2017-0014

[21] Tshomba Y Cavalli G. Priorities of biomedical research. Int J Cardiol. (2017) 245:256. doi: 10.1016/j.ijcard.2017.07.073 28755950

[22] van Wesel M. Evaluation by citation: trends in publication behavior, evaluation criteria, and the strive for high impact publications. Sci Eng Ethics. (2016) 22:199–225. doi: 10.1007/s11948-015-9638-0, PMID: 25742806 PMC4750571

[23] Miles M Huberman A Saldaña J. Qualitative Data Analysis—a Methods Sourcebook. 3rd ed. SAGE Publications. (2014). 78–80

[24] Clay M Hiraki LT Lamot L Medhat BM Sana S Small AR. Developing reflection and collaboration in translational medicine toward patients and unmet medical needs. Front Med (Lausanne). (2019) 6:94. doi: 10.3389/fmed.2019.00094, PMID: 31131280 PMC6509800

[25] Eley DS Jensen C Thomas R Benham H. What will it take? Pathways, time and funding: Australian medical students’ perspective on clinician-scientist training. BMC Med Educ. (2017) 17:242. doi: 10.1186/s12909-017-1081-2, PMID: 29216896 PMC5721615

[26] Tijdink JK Vergouwen ACM Smulders YM. Publication pressure and burn out among Dutch medical professors: a Nationwide survey. PLoS One. (2013) 8:e73381. doi: 10.1371/journal.pone.0073381, PMID: 24023865 PMC3762753

[27] Tijdink JK Verbeke R Smulders YM. Publication pressure and scientific misconduct in medical scientists. J Empir Res Hum Res Ethics. (2014) 9:64–71. doi: 10.1177/1556264614552421, PMID: 25747691

[28] Agrawal AA. Corruption of journal impact factors. Trends Ecol Evol. (2005) 20:157. doi: 10.1016/j.tree.2005.02.002 16701362

[29] Smith EM. Reimagining the peer-review system for translational health science journals. Clin Transl Sci. (2021):cts.13050. doi: 10.1111/cts.13050 PMC8301572 33963670

[30] Rubio DM Schoenbaum EE Lee LS Schteingart DE Marantz PR Anderson KE. Defining translational research: implications for training. Acad Med. (2010) 85:470–5. doi: 10.1097/ACM.0b013e3181ccd618, PMID: 20182120 PMC2829707

[31] Rietschel ET Bruckner-Tuderman L Schütte G Wess G. Translation—moving medicine forward faster. Sci Transl Med. (2015) 7 Editorial. doi: 10.1126/scitranslmed.aaa1470 25739759

[32] Cornfield DN Lane R Rosenblum ND Hostetter M Jobe A Albertine K. Patching the pipeline: creation and retention of the next generation of physician-scientists for child health research. J Pediatr Mosby. (2014) 165:882–884.e1. doi: 10.1016/j.jpeds.2014.07.037, PMID: 25441382

[33] Smith C Baveja R Grieb T Mashour GA. Toward a science of translational science. J Clin Transl Sci. (2017) 1:253–5. doi: 10.1017/cts.2017.14, PMID: 29657860 PMC5890312

[34] Ravenscroft J Liakata M Clare A Duma D. Measuring scientific impact beyond academia: an assessment of existing impact metrics and proposed improvements. PLoS One. (2017) 12:e0173152. doi: 10.1371/journal.pone.0173152, PMID: 28278243 PMC5344357

[35] Van Noorden R. Impact factor gets a heavyweight rival. Nature. (2016) 540:325–6. doi: 10.1038/nature.2016.21131, PMID: 27974784

[36] Bornmann L Marx W. How to evaluate individual researchers working in the natural and life sciences meaningfully? A proposal of methods based on percentiles of citations. Scientometrics. (2014) 98:487–509. doi: 10.1007/s11192-013-1161-y

[37] Alonso S Cabrerizo FJ Herrera-Viedma E Herrera F. Hg-index: a new index to characterize the scientific output of researchers based on the h- and g-indices. Scientometrics. (2010) 82:391–400. doi: 10.1007/s11192-009-0047-5

